# Yeast-produced RBD-based recombinant protein vaccines elicit broadly neutralizing antibodies and durable protective immunity against SARS-CoV-2 infection

**DOI:** 10.1038/s41421-021-00315-9

**Published:** 2021-08-18

**Authors:** Jinkai Zang, Yuanfei Zhu, Yu Zhou, Chenjian Gu, Yufang Yi, Shuxia Wang, Shiqi Xu, Gaowei Hu, Shujuan Du, Yannan Yin, Yalei Wang, Yong Yang, Xueyang Zhang, Haikun Wang, Feifei Yin, Chao Zhang, Qiang Deng, Youhua Xie, Zhong Huang

**Affiliations:** 1 CAS Key Laboratory of Molecular Virology & Immunology, Institut Pasteur of Shanghai, Chinese Academy of Sciences, University of Chinese Academy of Sciences, Shanghai, China; 2 Key Laboratory of Medical Molecular Virology (MOE/NHC/CAMS), Department of Medical Microbiology and Parasitology, School of Basic Medical Sciences, Shanghai Institute of Infectious Diseases and Biosecurity, Shanghai Medical College, Fudan University, Shanghai, China; 3 Key Laboratory of Tropical Translational Medicine of Ministry of Education, Hainan Medical University, Haikou, Hainan China; 4 Hainan Medical University-The University of Hong Kong Joint Laboratory of Tropical Infectious Diseases, Hainan Medical University, Haikou, Hainan China; 5 BSL-3 Laboratory of Fudan University, School of Basic Medical Sciences, Shanghai Medical College, Fudan University, Shanghai, China; 6 Children’s Hospital, Shanghai Medical College, Fudan University, Shanghai, China

**Keywords:** Immunology, Biological techniques

## Abstract

Massive production of efficacious SARS-CoV-2 vaccines is essential for controlling the ongoing COVID-19 pandemic. We report here the preclinical development of yeast-produced receptor-binding domain (RBD)-based recombinant protein SARS-CoV-2 vaccines. We found that monomeric RBD of SARS-CoV-2 could be efficiently produced as a secreted protein from transformed *Pichia pastoris* (*P. pastoris*) yeast. Yeast-derived RBD-monomer possessed functional conformation and was able to elicit protective level of neutralizing antibodies in mice. We further designed and expressed a genetically linked dimeric RBD protein in yeast. The engineered dimeric RBD was more potent than the monomeric RBD in inducing long-lasting neutralizing antibodies. Mice immunized with either monomeric RBD or dimeric RBD were effectively protected from live SARS-CoV-2 virus challenge even at 18 weeks after the last vaccine dose. Importantly, we found that the antisera raised against the RBD of a single SARS-CoV-2 prototype strain could effectively neutralize the two predominant circulating variants B.1.1.7 and B.1.351, implying broad-spectrum protective potential of the RBD-based vaccines. Our data demonstrate that yeast-derived RBD-based recombinant SARS-CoV-2 vaccines are feasible and efficacious, opening up a new avenue for rapid and cost-effective production of SARS-CoV-2 vaccines to achieve global immunization.

## Introduction

The pandemic of coronavirus disease 2019 (COVID-19), caused by the newly emerged severe acute respiratory syndrome coronavirus 2 (SARS-CoV-2), has created a worldwide health emergency^1–4^. SARS-CoV-2 possesses a ~30 kb single-stranded RNA genome^5,6^. Spike (S) protein mediates viral entry by engaging its host receptor, human angiotensin-converting enzyme 2 (hACE2), and triggering membrane fusion^7–10^. The ectodomain of S protein can be divided into two functionally distinct subunits, S1 and S2, which are responsible for receptor binding and membrane fusion, respectively. S1 can be further subdivided into N-terminal domain (NTD) and C-terminal domain (CTD). CTD comprises the receptor-binding domain (RBD) that directly interacts with the hACE2 receptor^11^.

Several types of SARS-CoV-2 vaccines have been approved for emergency use, including inactivated whole-virus vaccine, mRNA vaccine, adenovirus-vectored vaccine, and recombinant protein vaccine^12–14^. Most vaccines use or express SARS-CoV-2 S or its RBD as the vaccine antigen, because they are the main inducer of neutralizing antibodies^15–17^. In fact, insect cell- or mammalian cell-produced recombinant SARS-CoV-2 S or RBD protein vaccines have been shown to potently induce neutralizing antibodies in preclinical and clinical trials^12,13,18–20^. One of these recombinant protein-based candidate vaccines, the CHO cell-produced dimeric tandem-repeat RBD vaccine, has recently been authorized for emergency use in humans^12^. To contain the ongoing COVID-19 pandemic, massive immunization at a global scale is required. It is estimated that 16 billion doses of vaccine have to be made with majority of them being used in developing countries^21^. Despite the successful demonstration of the CHO cell-derived recombinant RBD vaccine, such a technology and its associated production facility are not available in most developing countries, calling for a more cost-effective and widely used recombinant expression system that may allow rapid adaptation by vaccine manufacturers in developing countries for local production of recombinant RBD protein vaccines.

Virus mutation and evolution pose a significant challenge to SARS-CoV-2 vaccine and therapeutics development. Some of the previously identified neutralizing monoclonal antibodies partially or completely lost neutralization potency toward the newly emerged SARS-CoV-2 variants, including the B.1.1.7 and B.1.351 strains^22,23^. In addition, plasma from convalescent patients or sera from volunteers immunized with Moderna SARS-Co-2 mRNA-1273 or Pfizer BNT162b2 vaccine showed significantly reduced neutralization activities against B.1.351^24^. Hence, for both existing vaccines and newly developed vaccine candidates, it is important to evaluate their abilities to elicit broadly neutralizing antibodies against circulating SARS-CoV-2 variants to ensure vaccine efficacy.

In the present study, we investigated the possibility to produce SARS-CoV-2 RBD-based recombinant protein vaccines in yeast, a cost-effective and robust recombinant vaccine production system for which the manufacture expertise and facility are available in some developing countries. We found that monomeric RBD of SARS-CoV-2 could be efficiently produced as a secreted protein from transformed *P. pastoris* yeast. Yeast-derived RBD-monomer was able to elicit neutralizing antibodies in mice. We further designed and expressed a genetically linked dimeric RBD protein in yeast. The engineered dimeric RBD was more potent than the monomeric RBD in inducing long-lasting neutralizing antibodies. Mice immunized with either monomeric RBD or dimeric RBD were effectively protected against live SARS-CoV-2 challenge even at 18 weeks after the last vaccine dose. More importantly, the antisera raised against the prototype strain-derived RBD could neutralize the two predominant circulating variants B.1.1.7 and B.1.351, indicating broad-spectrum protective potential of the RBD vaccines. Together, our data demonstrate yeast-derived, SARS-CoV-2 RBD-based recombinant protein vaccines are feasible and efficacious, opening up a new avenue for rapid and cost-effective production of large amounts of SARS-CoV-2 vaccine doses to allow massive immunization at a global scale.

## Results

### Production of recombinant monomeric RBD of SARS-CoV-2 in yeast

**Fig. 1:**
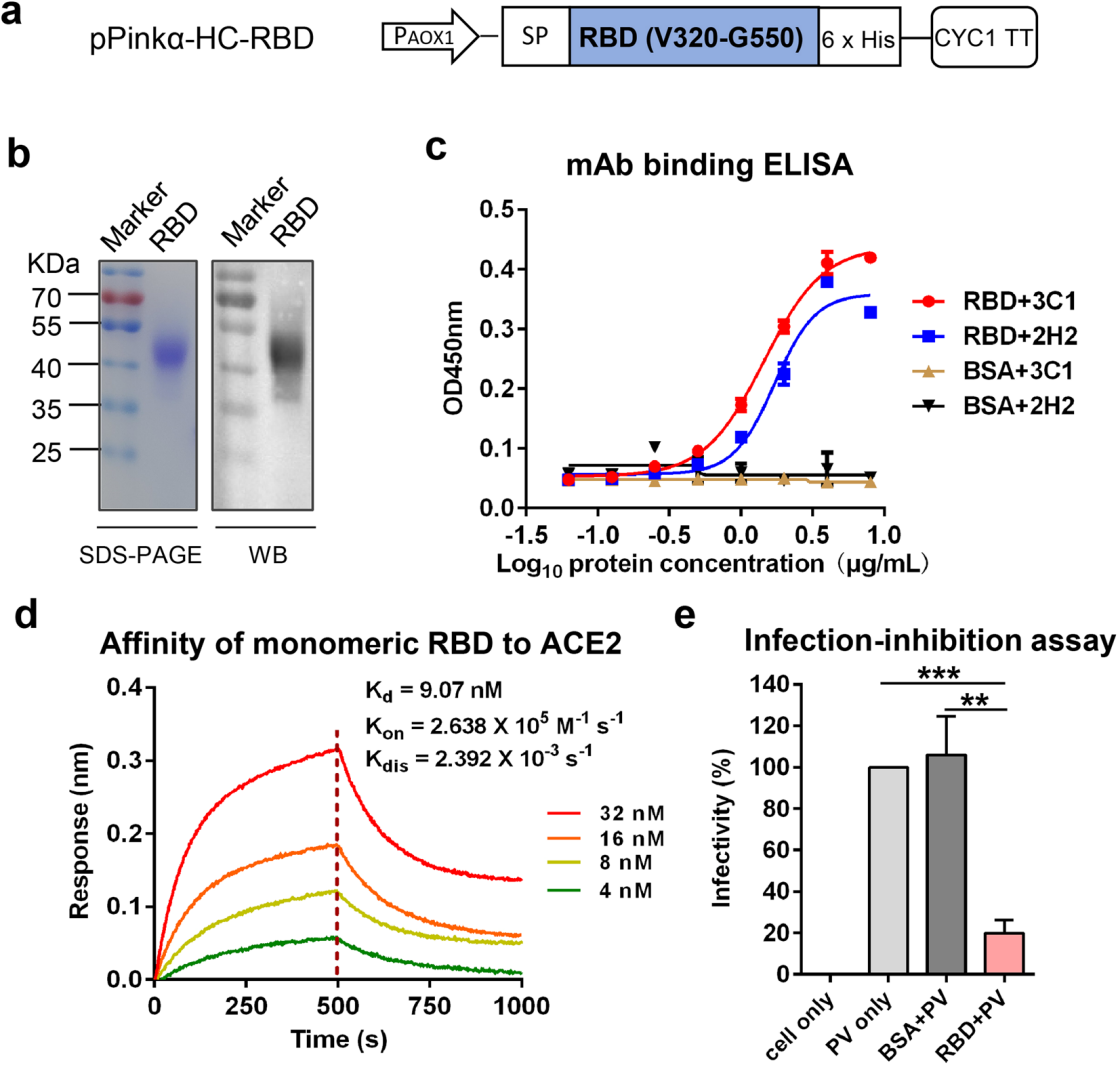
Production and characterization of recombinant monomeric RBD protein of SARS-CoV-2 in yeast. **a** Schematic diagram of the expression construct pPinkα-HC-RBD. P_AOX1_, AOX1 promoter; SP, signal peptide; CYC1 TT, CYC1 transcription termination region. **b** SDS-PAGE (left panel) and western blotting (WB; right panel) analysis of purified SARS-CoV-2 RBD protein. An anti-RBD (inclusion bodies) polyclonal antibody served as the detection antibody in WB assay. **c** Reactivity of yeast-derived RBD with neutralizing MAbs 2H2 and 3C1 determined by ELISA. BSA served as negative control. Data shown are means ± SEM of OD450 readings from triplicate wells. **d** Binding affinity of yeast-derived RBD to immobilized hACE2-Fc determined by BLI. RBD protein concentrations used were shown. **e** Inhibition of cell entry of SARS-CoV-2 pseudovirus (PV) by yeast-produced RBD protein. Data are means ± SEM of triplicate wells. Statistical significance was determined by Student’s *t*-test and indicated as follows: **P < 0.01; ***P < 0.001.

To produce SARS-CoV-2 RBD recombinant protein in yeast, an expression vector termed pPinkα-HC-RBD was constructed. This vector encoded SARS-CoV-2 RBD (residues 320–550) fused with an N-terminal α-mating factor signal peptide and a C-terminal 6× His-tag (Fig. 1a). The pPinkα-HC-RBD vector was used to transform *P. pastoris* yeast. The resulting yeast transformants were analyzed for the presence of RBD in culture supernatant by ELISA. Most of the yeast clones showed significant binding affinities (Supplementary Fig. S1a), indicating that recombinant RBD was expressed and secreted. One of the high expressors, clone # 8, was selected and used for subsequent antigen preparation. Recombinant RBD protein was purified from yeast culture supernatant as described in the Materials and Methods section. The purified RBD migrated as a ~50 kDa protein band on SDS-PAGE (Fig. 1b). The identity of the recombinant RBD protein was verified by western blot analysis with an RBD-specific polyclonal antibody (Fig. 1b). This observed molecular mass (~50 kDa) of yeast-derived RBD is much higher than the predicted molecular weight (~26 kDa) based on its amino acid sequence, suggesting possible glycosylation. Therefore, purified RBD was subjected to treatment with endoglycosidases endo H or PNGase F, which cleave within the chitobiose core of high mannose and some hybrid oligosaccharides from N-linked glycoproteins or remove all N-linked oligosaccharides from glycoproteins, respectively^25^. As shown in Supplementary Fig. S1b, the samples treated with either PNGase F or endo H produced protein bands of ~30 kDa, close to the predicted molecular weight of monomeric RBD. These results indicated that yeast-produced monomeric RBD is glycosylated with N-glycans.

We performed several assays to evaluate the folding and conformation of the yeast-derived monomeric RBD protein. First, recombinant RBD was analyzed by ELISA for its reactivity to neutralizing monoclonal antibodies (mAbs) 2H2 and 3C1 which recognize conformational epitopes within RBD^26^. The yeast-derived RBD reacted with both 2H2 and 3C1 antibodies in an antigen dose-dependent manner whereas the control antigen (BSA) failed to produce any reactivity regardless of the antigen dose (Fig. 1c), indicating that the RBD was properly folded to display conformational neutralizing epitopes. Second, we performed bio-layer interferometry (BLI) assay to determine whether yeast-derived RBD could bind to human ACE2 receptor. As shown in Fig. 1d, the binding affinity between yeast-derived RBD and ACE2 was determined to be 9.07 nM in equilibrium dissociation constants (*K*_D_) with *K*_on_ of 2.638 × 10^5^ M^−1^ s^−1^ and *K*_dis_ of 2.392 × 10^−3^ s^−1^. Third, we evaluated whether the recombinant RBD could inhibit SARS-CoV-2 pseudovirus infection of human ACE2-overexpressing HEK 293T (denoted as 293T-hACE2) cells by competing for cellular ACE2 receptor. As shown in Fig. 1e, the infectivity of SARS-CoV-2 pseudovirus was greatly reduced by treatment with the recombinant RBD but not by the control protein (BSA). Together, the above data demonstrate that yeast-derived RBD may have acquired conformations mimicking those displayed on the SARS-CoV-2 virion and is therefore a strong vaccine candidate aimed to elicit neutralizing or receptor-blocking antibodies.

### Yeast-derived monomeric RBD potently elicited protective neutralizing antibodies in mice

**Fig. 2:**
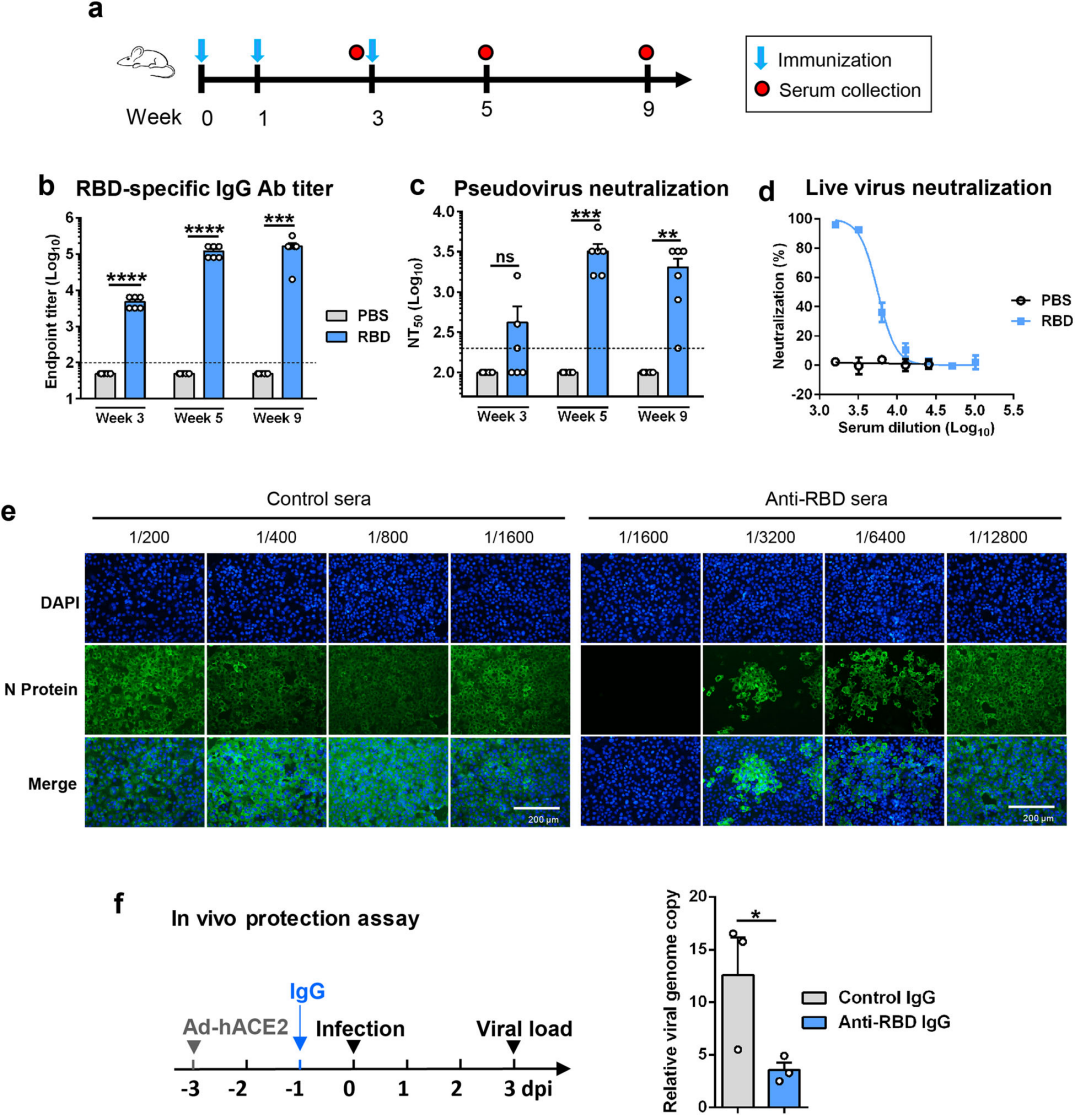
Immunization with yeast-derived monomeric RBD elicited protective antibodies in mice. **a** Immunization schedule. Groups of six BALB/c mice were injected i.p. with alum-formulated monomeric RBD protein (50 μg/dose) or PBS at weeks 0, 1, and 3. Serum samples were collected from individual mice at weeks 3, 5, and 9. **b** RBD-binding antibody (Ab) titers of the immune serum samples determined by ELISA. Binding titer below 1:100 (the lowest serum dilution; indicated by dashed line) was assigned a value of 1:50 for statistical analysis. **c** Measurement of neutralization activity of the antisera against SARS-CoV-2 pseudovirus. Serum samples that exhibited less than 50% neutralization at the lowest serum dilution (1:200; dashed line) were assigned a NT_50_ value of 100 for statistical analysis. Each symbol represents one mouse. **d**, **e** Neutralization activity of the pooled week-9 antisera against authentic SARS-CoV-2. Live SARS-CoV-2 was incubated with serially diluted antisera and then added to VeroE6 cells. After 48 h culture, the cells were subjected to qRT-PCR (**d**) or immunofluorescent staining analysis (**e**). Scale bars, 200 μm. **f** In vivo protective efficacy of IgG antibody purified from the week-9 antisera against authentic SARS-CoV-2 infection. Left panel: study outline. Ad5-hACE2, recombinant adenovirus 5 expressing human ACE2. Right panel: qPCR results are shown as viral RNA levels relative to GAPDH, using 2^−ΔCt^ method. Each symbol represents one mouse. Data in panels **b**, **c**, **d**, and **f** are expressed as means ± SEM. Statistical significance was determined by Student’s *t*-test and indicated as follows: ns, not significant; *P < 0.05; **P < 0.01; ***P < 0.001; ****P < 0.0001.

To rapidly evaluate the immunogenicity of yeast-produced monomeric RBD, a group of BALB/c mice (*n* = 6) were immunized by intraperitoneal (i.p.) injection with a relatively high dose of the candidate vaccine (50 μg of monomeric RBD formulated with Alum adjuvant) at weeks 0, 1, 3 and another group of mice were injected with PBS plus Alum adjuvant, serving as the control (Fig. 2a). Serum samples were collected at weeks 3, 5, and 9 and analyzed for RBD-specific antibody by ELISA. None of the serum samples from the control group exhibited any significant binding activity even at the lowest serum dilution tested (1:100); in contrast, RBD-binding activity was readily detectable in the serum samples from RBD-immunized mice at week 3, with geometric mean titer (GMT) of 4525, and the RBD-specific antibody titers increased greatly at week 5 (GMT = 113,137) and maintained at high levels (GMT = 126,992) at week 9 (Fig. 2b).

The antisera were assessed for their abilities to neutralize SARS-CoV-2 pseudovirus (retrovirus pseudotyped with the SARS-CoV-2 S protein). None of the serum samples from the PBS control group had neutralization activity even at the lowest serum dilution tested (1:200) (Fig. 2c). In contrast, for the RBD-immunized mice, three out of six antisera collected at week 3 showed measurable neutralization activity, and all antisera collected at week 5 and week 9 were able to neutralize pseudovirus infection with 50% neutralization titers (NT_50_s) ranging from 1600 to 6400 and from 200 to 3200, respectively (Fig. 2c). We also evaluated the antisera for their ability to block RBD binding to hACE2 in vitro. We found that the anti-RBD sera but not the control sera could dose-dependently block the interaction between RBD and hACE2 with calculated BT_50_ (50% blocking titer) values of 250 and 89 for the week-5 and week-9 anti-RBD sera, respectively. For the anti-RBD sera, their BT_50_ titers appeared to correlate well (*P* = 0.0087) with their NT_50_ against the SARS-CoV-2 pseudovirus (Supplementary Fig. S2), suggesting that blockade of hACE2 interaction with RBD exposed on the virion surface is likely the main neutralization mechanism for the RBD vaccine sera.

The week-9 antisera for each group were pooled and tested for their capacity to neutralize authentic SARS-CoV-2 virus (strain nCoV-SH01). As shown in Fig. 2d, e, the pooled anti-RBD sera were found to dose-dependently inhibit authentic SARS-CoV-2 infection with a calculated NT_50_ of 5645, whereas the control sera did not show any significant neutralization activity even at the sera dilution of 1:100 (the lowest dilution tested).

To assess the protective potential of the neutralizing antibodies, IgG was purified from the pooled anti-RBD and the control sera, respectively. Two groups (*n* = 3 per group) of naive mice were transduced with adenovirus 5 expressing human ACE2 (Ad5-hACE2) and 2 days later injected i.p. with anti-RBD IgG or control IgG, respectively. The recipient mice were challenged with live SARS-CoV-2 one day after IgG transfer, and analyzed for viral loads in the lungs by qRT-PCR at day 3 after viral infection. As shown in Fig. 2f, the viral loads in the lungs of the mice pretreated with anti-RBD IgG were significantly lower than those of the mice injected with the control IgG, indicating that the anti-RBD antibodies conferred partial protection in vivo.

Collectively, the above data demonstrate that yeast-derived monomeric RBD is capable of effectively eliciting protective antibody response in mice.

### Effects of antigen dose and adjuvant on immunogenicity of yeast-derived RBD- monomer

**Fig. 3:**
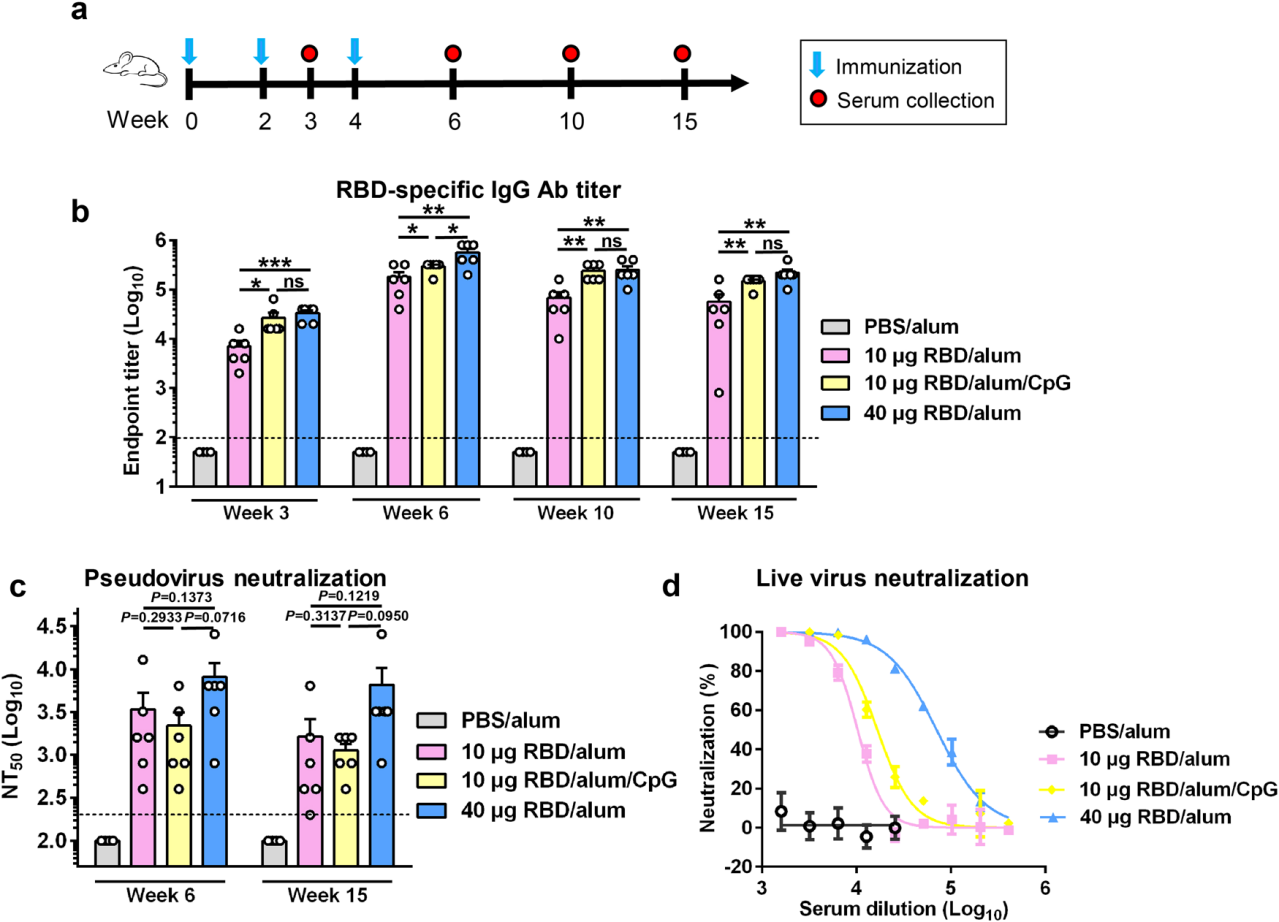
Effects of antigen dose and adjuvant on immunogenicity of monomeric RBD. **a** Immunization schedule. Four groups of BALB/c mice were immunized with alum-formulated PBS (PBS/alum), 10 μg of RBD formulated with alum (10 μg RBD/alum), 10 μg of RBD formulated with alum and CpG (10 μg RBD/alum/CpG), or 40 μg RBD/alum at weeks 0, 2, and 4. Antiserum samples were collected at weeks 3, 6, 10, and 15. **b** RBD-specific serum antibody titers determined by ELISA. Binding titer below the lowest serum dilution (1:100; dashed line) was assigned a value of 50. **c** Neutralizing antibody titers (NT_50_) of the antisera against SARS-CoV-2 pseudovirus. NT_50_ below the lowest serum dilution (1:200; dashed line) was assigned a value of 100. Each symbol represents one mouse. **d** Neutralization activity of the pooled week-6 antisera against authentic SARS-CoV-2. Data are presented as means ± SEM. *P* values were analyzed with unpaired *t*-test and indicated as follows: ns, not significant; *P < 0.05; **P < 0.01; ***P < 0.001.

Knowing that yeast-derived monomeric RBD is an antigen able to induce neutralizing antibodies, we therefore performed another mouse immunization experiment to evaluate different RBD vaccine formulations. Four groups of BALB/c mice (*n* = 6 per group) were immunized three times at a 2-week interval with either the control (PBS plus Alum) or one of the three experimental vaccine formulations, including 10 μg RBD plus Alum, 10 μg RBD plus Alum and CpG, and 40 μg RBD plus Alum (Fig. 3a). Serum samples from individual mice were collected at the indicated time points for antibody measurement (Fig. 3a). As shown in Fig. 3b, all the mice in the three vaccine groups had already developed considerable levels of RBD-specific antibodies at week 3 (1 week after the second immunization) and the antibody titers further increased at week 6 (2 weeks after the third immunization); in contrast, neither the week-3 nor week-6 serum samples from the control (PBS plus Alum) group exhibited RBD-binding activity. In addition, it was found that, in the presence of the same Alum adjuvant, the high antigen dose (40 μg) induced higher levels of RBD-specific antibodies than the low antigen dose (10 μg) at both week-3 and week-6, indicating that antigen dose is a critical determinant of vaccine immunogenicity. Moreover, at week 6, 100% of mice in the high-dose group had detectable levels of hACE2-binding blockade antibodies compared to only 50% in the low-dose group, and BT50s of the high-dose group were significantly higher than those of the low-dose group. Thus, there is a dose-dependent response for blocking antibody titers in mice (Supplementary Fig. S3a). The RBD-specific total IgG antibody titers in the mice immunized with 10 μg RBD plus Alum and CpG were only slightly higher than those in the mice receiving 10 μg RBD plus Alum at week 3, and comparable to those in the mice receiving 10 μg RBD plus Alum at week 6 (Fig. 3b). However, the ratios of RBD-specific IgG1 vs IgG2a for these two groups were significantly different (Supplementary Fig. S3b). Specifically, the antisera from the “10 μg RBD+Alum+CpG” group had lower IgG1/IgG2a ratios than those from the “10 μg RBD+Alum” group (Supplementary Fig. S3b), suggesting that the addition of CpG promotes type I T helper (Th) cell response bias, in agreement with previous findings^27,28^. The RBD-specific antibody titers of the three vaccine groups slightly dropped at weeks 10 and 15 as compared to those of the corresponding week-6 antisera, but still maintained at high levels (Fig. 3b).

We selected the week-6 and week-15 antisera for measurement of their neutralization capacity by SARS-CoV-2 pseudovirus neutralization assays. None of the sera from the control group showed neutralization regardless of the serum dilutions tested, whereas all of the sera from the three vaccine groups exhibited neutralization activity to different extents (Fig. 3c). Specifically, the geometric mean NT_50_ titers for the “10 μg RBD+Alum”, “10 μg RBD+Alum+CpG”, and “40 μg RBD+Alum” groups were 1796, 1425, and 5080, respectively, at week 6, and were 800, 1008, and 3592, respectively, at week 15 (Fig. 3c).

The week-6 antisera were pooled for each group and then tested for neutralization of authentic SARS-CoV-2. Based on immunofluorescence assay (IFA) results, the pooled “10 μg RBD+Alum”, “10 μg RBD+Alum+CpG”, and “40 μg RBD+Alum” antisera could fully prevent SARS-CoV-2 infection at serum dilutions up to 1:1600, 1:3200, and 1:6400, respectively, whereas the control sera were non-neutralizing even at the dilution of 1:200 (Supplementary Fig. S3c). qRT-PCR analysis showed that the calculated NT_50_s for the “10 μg RBD+Alum”, “10 μg RBD+Alum+CpG”, and “40 μg RBD+Alum” antisera were 10,296, 16,646, and 70,395, respectively (Fig. 3d).

### Design and expression of SARS-CoV-2 RBD-dimers in yeast

**Fig. 4:**
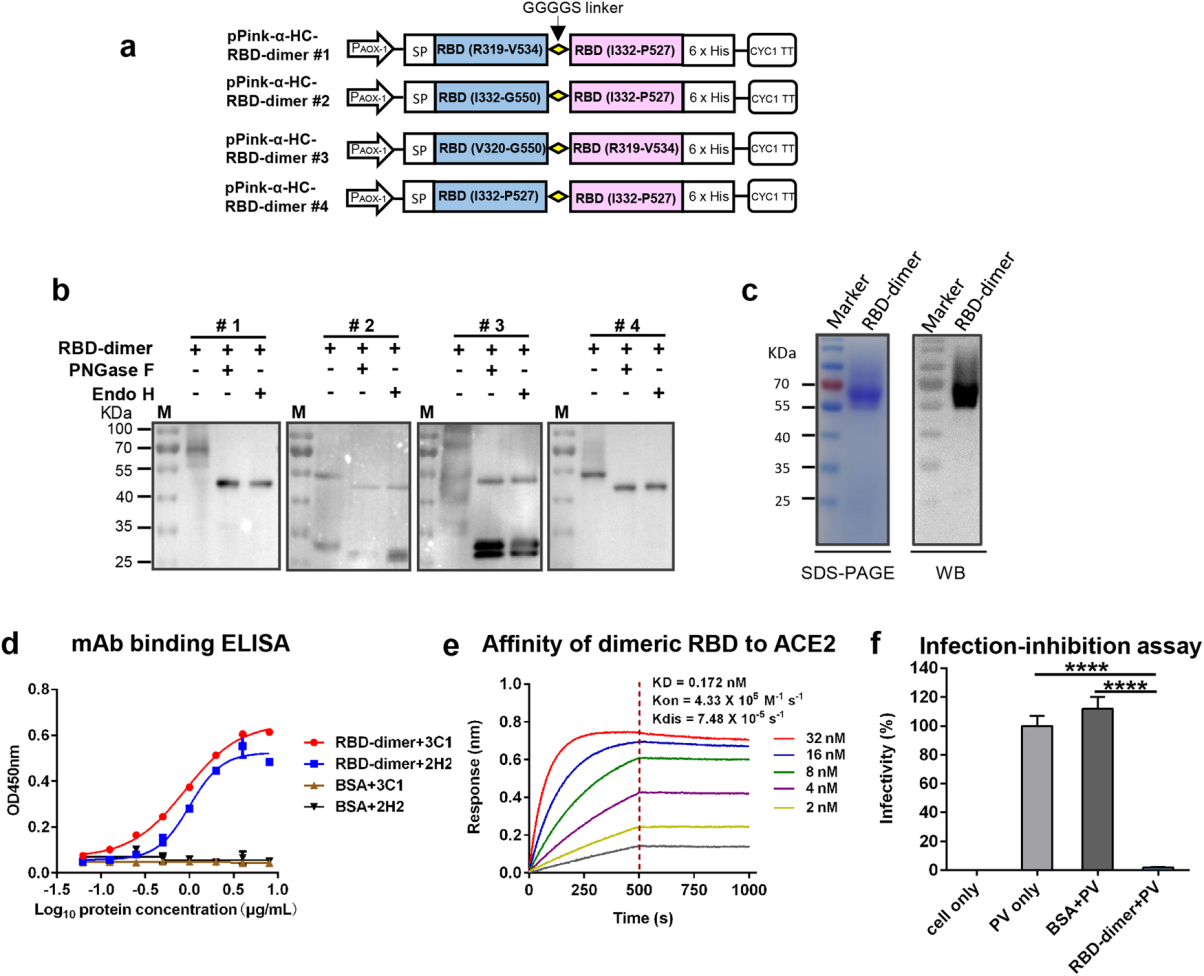
Production and characterization of recombinant SARS-CoV-2 RBD-dimer in yeast. **a** Schematic diagram of four expression vectors (designated #1 to #4) encoding tandem-repeat RBD-dimer. **b** Supernatants of each vector-transformed yeast cultures were digested with either endo H or PNGase F, and then subjected to western blotting analysis with an anti-RBD (inclusion bodies) polyclonal antibody. Symbol (+) indicates presence; (−) indicates absence. M, protein marker. **c** RBD-dimer protein was purified from culture supernatant of a yeast clone transformed with vector #1 and then analyzed by SDS-PAGE (left panel) and western blotting (WB; right panel) with an anti-RBD (inclusion bodies) polyclonal antibody. **d** Reactivity of RBD-dimer with neutralizing MAbs 2H2 and 3C1 measured by ELISA. BSA served as negative control. Data shown are means ± SEM of triplicate wells. **e** Binding affinity of yeast-derived dimeric RBD to immobilized hACE2-Fc determined by BLI. RBD-dimer protein concentrations tested were shown. **f** Inhibition of cell entry of SARS-CoV-2 pseudovirus (PV) by dimeric RBD protein. Data are means ± SEM of triplicate wells. Statistical was determined by Student’s *t*-test and indicated as follows: ns, not significant; ****P < 0.0001.

It was recently reported that a tandem-repeat RBD-dimer produced in mammalian cells possessed improved immunogenicity and neutralizing antibody-inducing ability as compared to monomeric RBD^12^. We therefore asked whether RBD-dimers could be designed and produced in yeast. Four yeast expression vectors were constructed, each encoding a fusion protein of two RBDs of varied lengths (Fig. 4a), and used to separately transform yeast. Western blot analysis of the culture supernatant of yeast transformants revealed that the #1 and #4 constructs produced predominantly single protein bands of ~70 and ~55 kDa, respectively, whereas two or multiple bands were observed for the #2 and #3 constructs (Fig. 4b). Following treatment with either endo H or PNGase F, single bands of ~47 kDa (which is close to the predicated molecular weight of RBD-dimers) was evident for the #1 and #4 constructs; in contrast, additional bands of ~27 kDa (likely representing monomeric RBD) were detected in the samples transformed with the #2 and #3 constructs, suggesting their expression products were partially cleaved to yield RBD-monomers (Fig. 4b). Although the #1 and #4 constructs yielded similar results in biochemical analysis, the antigen encoded by the #1 construct has additional amino acids at both N-terminal and C-terminal of its first RBD (R319-V534) as compared with that of the #4 construct (I332-P527), and therefore may contain more neutralizing epitopes or better preserve immunogenic conformation. Hence, we selected the #1 construct for preparation of RBD-dimer and subsequent immunological analyses.

Purified RBD-dimer was observed as a ~70 kDa band in SDS-PAGE and western blot (Fig. 4c) whereas endo H- or PNGase F-digested RBD-dimer migrated as single bands of ~47 kDa (Supplementary Fig. S4a), indicating that the dimeric RBD is also glycosylated. Size exclusion chromatography analysis confirmed that the purified dimeric RBD existed as dimer in solution with 93.3% purity (Supplementary Fig. S4b). The yeast-derived dimeric RBD reacted with both 2H2 and 3C1 antibodies in an antigen dose-dependent manner (Fig. 4d). The RBD-dimer exhibited high affinity toward hACE2 with *K*_D_ value being 0.172 nM (Fig. 4e), suggesting that it is more efficient in binding hACE2 receptor than the RBD-monomer (*K*_D_ = 9.07 nM, Fig. 1d). In SARS-CoV-2 pseudovirus infection inhibition assay, pretreatment with 60 ng/μL of RBD-dimer almost completely abolished pseudovirus infectivity (Fig. 4f). These data show that yeast-derived RBD-dimer was properly folded to display conformation-dependent neutralizing epitopes and receptor-binding site.

### Yeast-derived RBD-dimer is highly immunogenic and protective in mice

**Fig. 5:**
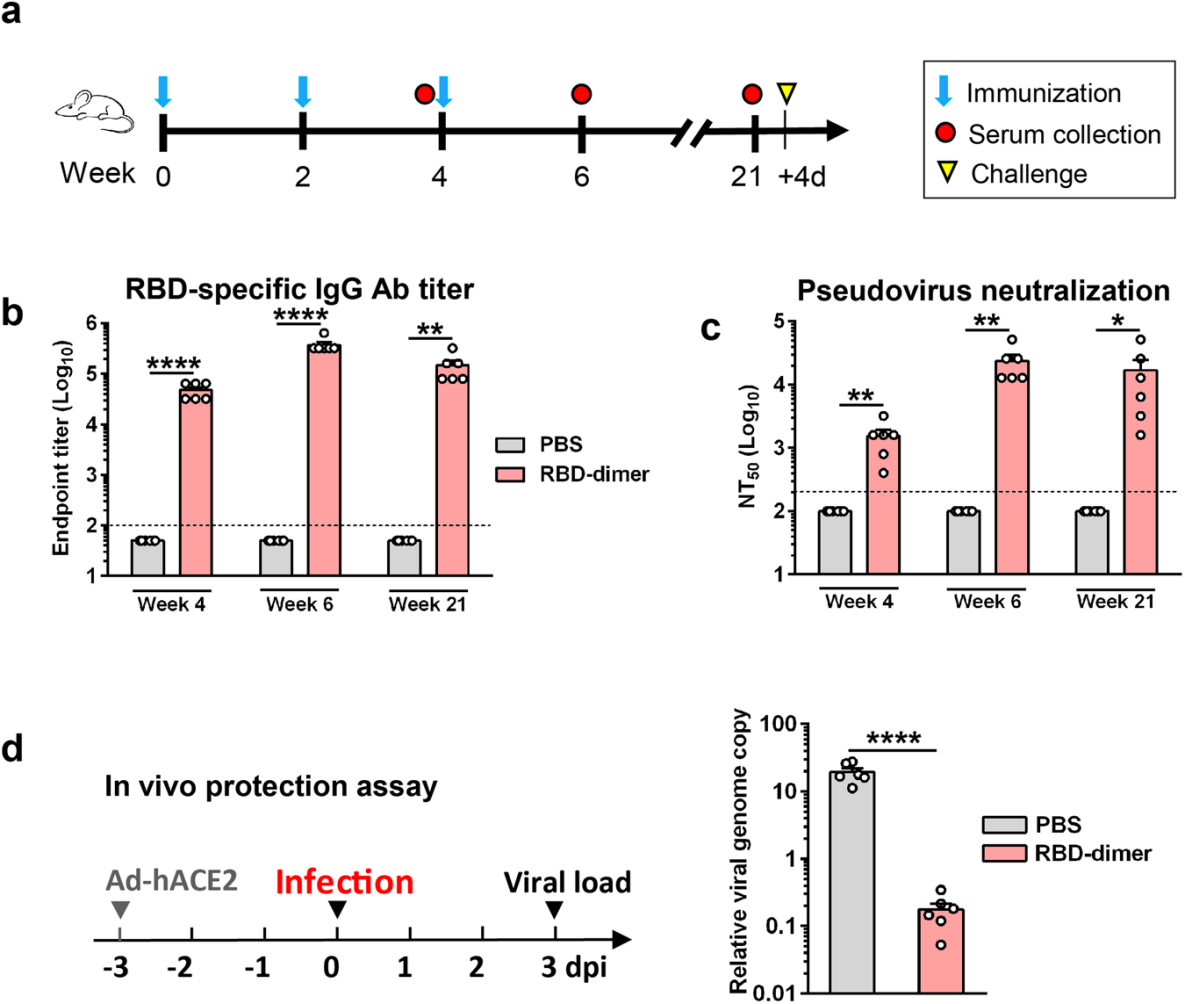
Yeast-derived RBD-dimer elicited durable protective antibodies in mice. **a** Schedule of mouse immunization and challenge experiments. Two groups of six BALB/c mice were immunized with alum-formulated RBD-dimer protein (5 μg/dose) or PBS at weeks 0, 2, and 4. Serum samples were taken at weeks 4, 6, and 21. Four days after the last serum collection, the mice were challenged with live SARS-CoV-2. **b** RBD-specific antibody titers in the antisera were measured by ELISA. Anti-PBS sera did not show any binding activity at the lowest serum dilution (1:100; dashed line) and was assigned a titer of 50. **c** Neutralizing antibody titers of the antisera against SARS-CoV-2 pseudovirus. Anti-PBS sera did not show any neutralization at the lowest serum dilution (1:200; dashed line) and was assigned a NT_50_ value of 100. **d** Active immunization with RBD-dimer provided protection against live SARS-CoV-2 infection. Left panel: study outline. After transduction with Ad5-hACE2, the vaccinated mice were challenged with live SARS-CoV-2 and then analyzed for viral loads in the lungs by qRT-PCR. Right panel: qPCR results are shown as viral RNA levels relative to GAPDH, using 2^−ΔCt^ method. Each symbol represents one mouse. For panels **b**–**d**, data are presented as means ± SEM. *P* values were analyzed with unpaired *t*-test and indicated as follows: *P < 0.05; **P < 0.01; ****P < 0.0001.

To evaluate the immunogenicity of yeast-produced dimeric RBD, we performed a mouse immunization experiment. Two groups of BALB/c mice (*n* = 6 per group) were injected i.p. with 5 μg of RBD-dimer plus Alum adjuvant or with PBS plus Alum, respectively, at weeks 0, 2, 4, and blood draws were performed at weeks 4, 6, and 21 (Fig. 5a). None of the serum samples from the control (PBS plus Alum) mice showed detectable RBD-binding activity even at the lowest serum dilution tested (1:100); in contrast, RBD-binding antibody titers of the RBD-dimer-immunized mice were readily detectable (GMT = 45,255) at week 4, peaked (GMT = 359,188) at week 6, and remained at high levels (GMT = 126,992) at week 21 (Fig. 5b). Results from pseudovirus neutralization assays revealed that the serum samples from the RBD-dimer-immunized mice possessed potent neutralization activities, with geometric mean NT_50_s of 1270, 20,319, and 9051, for the week-4, week-6, and week-21 antisera, respectively (Fig. 5c). We also compared NT_50_s with endpoint titers of individual serum samples from the RBD-dimer-immunized mice, and the results shows a positive correlation between them (Supplementary Fig. S5).

To assess the RBD-dimer vaccine’s protective efficacy, all mice in the control and the RBD-dimer vaccine groups were transduced with Ad5-hACE2 and subsequently challenged with live SARS-CoV-2 (Fig. 5d, left). The viral loads in the lungs of the RBD-dimer-immunized mice were significantly lower than those of the control mice by ~120-fold (Fig. 5d, right), indicating that the RBD-dimer vaccine can protect mice against live SARS-CoV-2 infection.

### RBD-dimer is more potent than RBD-monomer in eliciting neutralizing antibodies

**Fig. 6:**
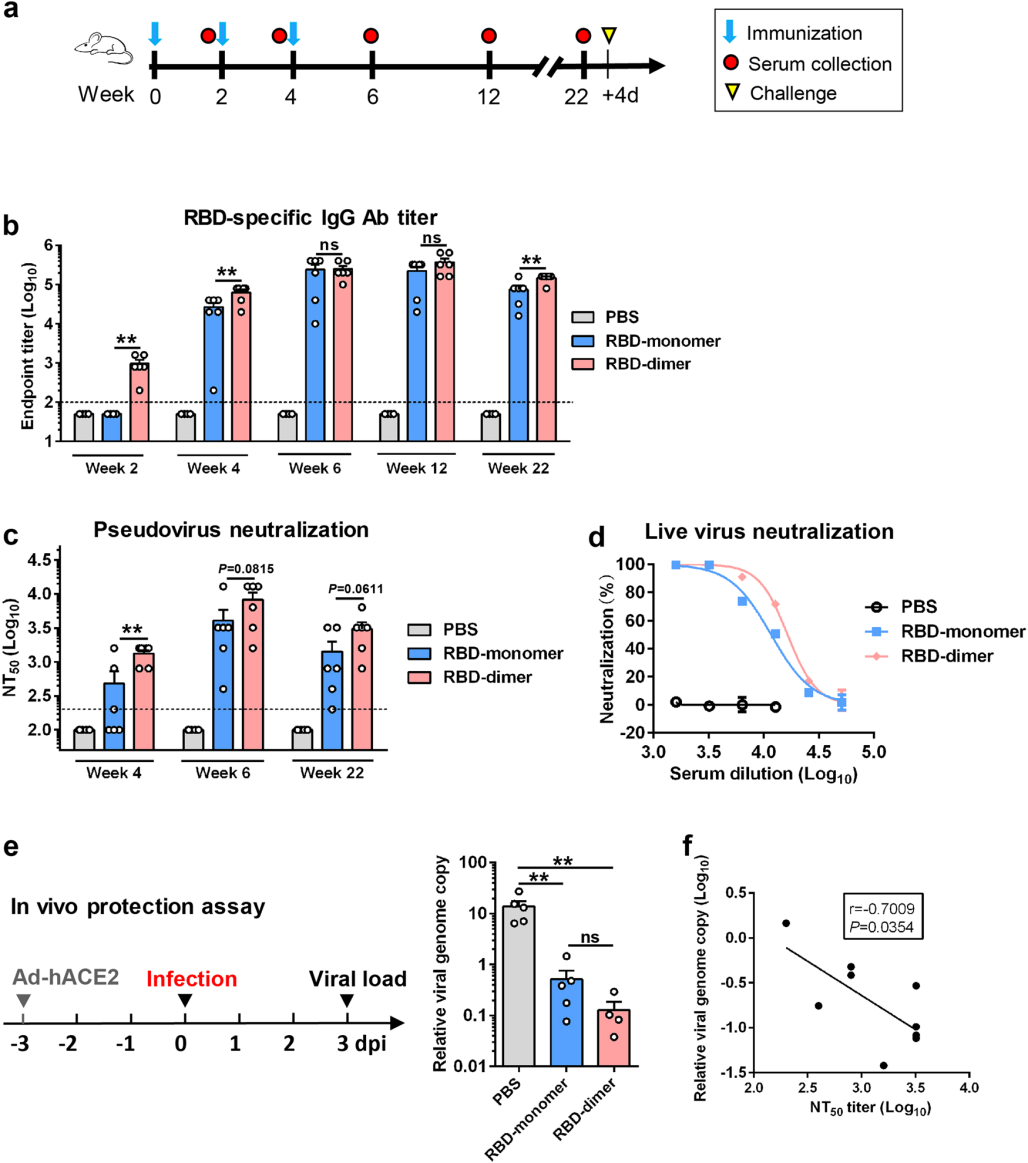
Direct comparison of immunogenicity and protective efficacy was made between RBD-monomer and -dimer proteins. **a** Schedule of mouse immunization and challenge experiments. Three groups of BALB/c mice were immunized with alum-formulated RBD-monomer (10 μg/dose), RBD-dimer protein (10 μg/dose), or PBS at weeks 0, 2, and 4. Serum samples were harvested at weeks 2, 4, 6, 12, and 22. All mice were challenged with live SARS-CoV-2 at 4 days after the last blood draw. **b** The antisera were analyzed for RBD-binding antibody titers by ELISA. PBS immune sera did not exhibit any binding activity at the lowest dilution (1:100; dashed line) and was denoted as a titer of 50. **c** Neutralizing antibody titers of the antisera against SARS-CoV-2 pseudovirus. Anti-PBS sera did not confer any neutralization at the lowest dilution (1:200; dashed line) and was denoted as a NT_50_ value of 100. Each symbol represents one mouse. **d** Neutralization activity of the pooled week-6 immune sera against live SARS-CoV-2. **e** In vivo challenge assay. Immediately after the last blood draw at week 22 (see panel **a**), the vaccinated mice were transduced with Ad5-hACE2 and 3 days later challenged with live SARS-CoV-2. Mouse lungs were collected 3 days post infection and analyze for viral loads by qRT-PCR. Left panel: study outline. Right panel: qPCR results are shown as viral RNA levels relative to GAPDH, using 2^−ΔCt^ method. Each symbol represents one mouse. **f** The negative correlation between pulmonary viral load and serum neutralizing antibody titers. In panels **b**–**e**, data are means ± SEM of triplicate wells. In panels **b**, **c**, and **e**, *P* values were analyzed with unpaired *t*-test and indicated as follows: ns, not significant; **P < 0.01.

To directly compare the immunogenicity of RBD-monomer and RBD-dimer, groups of BALB/c mice (*n* = 6 per group) were immunized with equal amount of antigen (10 μg of RBD-monomer or -dimer) formulated with Alum at weeks 0, 2, and 4, respectively (Fig. 6a). Another group of mice were injected with PBS plus Alum, serving as the control. As shown in Fig. 6b, all of the RBD-dimer-immunized mice developed RBD-specific serum antibodies after the prime (week 2) whereas none of the mice in the RBD-monomer vaccine group showed detectable RBD-specific antibody response, indicating that the RBD-dimer is able to elicit antigen-specific antibody response more rapidly than the RBD-monomer. At week 4, the level of RBD-binding antibodies in the mice of the RBD-dimer group remained higher than that of the RBD-monomer group (GMT = 56,569 vs 13,128) (Fig. 6b). The RBD-binding antibody titers of both vaccinated groups further elevated after the second booster and reached comparable levels (GMT = 224,492 vs 131,284) at week 6 (Fig. 6b).

The antisera collected at weeks 4 and 6 were assessed for neutralization of SARS-CoV-2 pseudovirus. As shown in Fig. 6c, the antisera from the RBD-dimer group in general exhibited higher neutralization activities than those from the RBD-monomer group. Specifically, the geometric mean NT_50_s were 1270 and 252 for the anti-RBD-dimer and anti-RBD-monomer, respectively, at week 4, and were 6400 and 2540, respectively, at week 6. The control (PBS) sera collected at both time points did not show neutralization effect.

The week-6 antisera were pooled for each group and tested for neutralization of authentic SARS-CoV-2. Both the anti-RBD-dimer and anti-RBD-monomer pooled sera could neutralize authentic SARS-CoV-2 infection in an antisera dose-dependent manner with calculated NT_50_ of 16,453 and 11,674, respectively (Fig. 6d), whereas no neutralization was observed for the control sera (Fig. 6d; Supplementary Fig. S6).

### Yeast-derived RBD vaccines elicited sustained protective immunity

To determine whether the neutralizing antibody responses induced by the candidate vaccines are durable, the three groups of mice were maintained until ~23 weeks after the first immunization. Blood draws were performed at weeks 12 and 22 and the sera obtained were analyzed by ELISA and pseudovirus neutralization assays. As shown in Fig. 6b, c, the anti-RBD-monomer and anti-RBD-dimer sera collected at week 12 exhibited RBD binding and neutralization at levels comparable to those of the corresponding week-6 antisera. Both the RBD-binding titers and NT_50_s of the serum samples collected at week 22 were lower than those of the corresponding week-6 antisera (Fig. 6b, c) but still remained at high levels. Specifically, the NT_50_s of the week-22 anti-RBD-dimer sera ranged from 800 to 6400 (geometric mean NT_50_ = 2540), while the NT_50_s of the anti-RBD-monomer sera were only from 200 to 3200 (geometric mean NT_50_ = 898).

One day after the last blood collection, the three groups of mice were inoculated intranasally with Ad5-hACE2 and 3 days later challenged with authentic SARS-CoV-2. The viral loads in the lungs of the challenged mice were determined by qRT-PCR analysis. The results showed that the viral loads detected in the RBD-monomer- or RBD-dimer-immunized mice were lower than those in the control mice by 40- and 120-folds, respectively (Fig. 6e), demonstrating high protective efficacy by the RBD-based vaccines. The viral loads of individual mice appeared to inversely correlate with the NT_50_ titers of the corresponding mice before challenge (Fig. 6f), suggesting that neutralizing antibody plays a key role in protective immunity against SARS-CoV-2.

### Antisera elicited by the yeast-derived RBD vaccines efficiently neutralized B.1.1.7 and B.1.351 variants

**Fig. 7:**
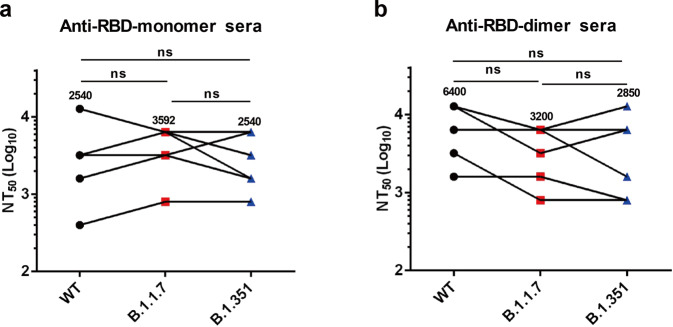
Antisera induced by the yeast-derived monomeric and dimeric RBD vaccines efficiently neutralized SARS-CoV-2 variants. Neutralizing titers of week-6 anti-RBD-monomer (**a**) and anti-RBD-dimer sera (**b**) against B.1.1.7 and B.1.351 variants were measured using pseudovirus neutralization assay. For comparison, NT50s of antisera against WT pseudovirus (Fig. 6c) were also shown. Geometric means were calculated for each data set and shown. Each symbol represents one mouse. Statistical significance was determined by Student’s *t*-test and indicated as follows: ns, not significant.

The individual week-6 antisera of the control, RBD-monomer and RBD-dimer groups were evaluated for their neutralization breadth by using retroviruses pseudotyped with the spike of the B.1.1.7 or B.1.351 variants. All antisera from the RBD-monomer group remained highly effective against the B.1.1.7 and B.1.351 pseudoviruses with NT_50_s between 800 and 6400 (geometric mean NT_50_ = 3592) and between 800 and 6400 (geometric mean NT_50_ = 2540), respectively (Fig. 7a). The NT_50_s of antisera from the RBD-dimer group against the B.1.1.7 and B.1.351 variants ranged from 800 to 6400 (geometric mean NT_50_ = 3200) and from 800 to 12,800 (geometric mean NT_50_ = 2850), respectively (Fig. 7b). Overall, the neutralization potency of the anti-RBD-monomer and anti-RBD-dimer sera toward B.1.1.7 or B.1.351 was similar to those against the original strain (geometric mean NT_50_s of 2540 and 6400, respectively) (Fig. 7). These results show that the antisera raised against recombinant RBDs of a single original strain remained effective against the predominant circulating SARS-CoV-2 variants, thus demonstrating their broad-spectrum neutralization capacity and protective potential.

## Discussion

The present study aimed to investigate the possibility of producing SARS-CoV-2 RBD-based recombinant protein vaccines in yeast. It was found that either RBD-monomer or tandem-repeat RBD-dimer could be expressed as secreted proteins in *P. pastoris* yeast and these proteins could induce broadly neutralizing antibodies and durable protective immunity in mice.

Yeast is a robust, highly scalable, and cost-effective system for recombinant protein vaccine production^29,30^. Since the introduction of yeast-derived HBV recombinant vaccines, yeast-based vaccine production platforms have been established in many developing countries. In the present study, we found that monomeric RBD could be produced in transgenic *P. pastoris* yeast at levels up to 200 mg/L under laboratory conditions while the expression levels of dimeric RBD were about 5–10-folds lower. We believe that the yields could be further improved by optimization of regulatory elements and through high-density yeast fermentation. Given the availability of facility and expertise for yeast-based vaccine manufacture in many developing countries, the technology of yeast-derived RBD-based SARS-CoV-2 vaccines could be readily transferred to developing countries, allowing rapid production and deployment of large amounts of vaccines locally in order to better control the global COVID-19 pandemic.

In the present study, we found that yeast-derived dimeric RBD were more potent than monomeric RBD in eliciting neutralizing antibodies (Fig. 6c). Specifically, the geometric mean NT_50_s for the RBD-monomer- and the RBD-dimer-elicited antisera were 252 and 1270 at week 4, 2540 and 6400 at week 6, and 898 and 2540 at week 22, respectively (Fig. 6c). Our data are in agreement with the results from a recently published study, in which the immunogenicities of mammalian cell-produced RBD-monomer and -dimer were compared^12^. Because the yeast-derived RBD-dimer showed higher affinity to the hACE2 receptor than did the yeast-derived RBD-monomer (Figs. 1d and 4e), it is therefore likely that better folding and exposure of RBM (the main target of potent neutralizing monoclonal antibodies) on the RBD-dimer may have contributed to the superior vaccine potency of the RBD-dimer. It is also possible that, compared with the RBD-monomer, the RBD-dimer might trigger enhanced uptake and presentation by antigen-presenting cells or induce stronger antigen-specific germinal center responses due to its increased size^31^. However, this hypothesis remains to be verified. Nonetheless, we found that both yeast-derived RBD-monomer and -dimer could induce long-lasting neutralizing antibody responses which protected mice against viral challenge even at 18 weeks after the last immunization (Fig. 6e). Our data suggest that the yeast-derived RBD-monomer and -dimer are viable vaccine candidates worthy of further testing in nonhuman primates and in clinical trials. In support of our notion, a recently developed SARS-CoV-2 vaccine candidate (RBD219-N1C1), which is based on yeast-produced monomeric RBD^32^, is now being tested in a Phase I/2 clinical trial in India (CTRI Number: CTRI/2020/11/029032; http://ctri.nic.in/Clinicaltrials/pdf_generate.php?trialid=48329&EncHid=&modid=&compid=%27,%2748329det%27).

In this study, we found that yeast-derived SARS-CoV-2 RBD protein was highly glycosylated (Supplementary Figs. S1 and S4). Compared to mammalian cell- and insect cell-produced RBDs^12,13^, yeast-derived RBDs appeared to have a distinct glycosylation pattern. For mammalian cell-produced RBD, N-glycans are attached to residues N331 and N343, while O-glycans are linked to residues T323 and S325^33^. Specifically, CHO cell-derived RBD protein contains complex N-glycans and mainly Core-1 O-glycans^34^. We did not determine the glycan types on the yeast-expressed RBD. However, previous studies show that, for *P. pastoris*-derived proteins, N-glycans are high-mannose type without core fucose^35,36^, while O-glycans are linear chains of four to five α-linked mannose residues^37^. Thus, it is likely that the glycostructures of yeast-expressed RBDs differ from those of CHO-produced RBDs. The difference in glycosylation between yeast- and CHO-produced SARS-CoV-2 RBDs appeared not to influence their immunogenicity. For example, 10 μg/dose of CHO-produced RBD-dimer induced potent antibody responses in BALB/c mice with NT50s against live virus ranging from 512 to >4096 after the second (last) immunization^12^, while in our study the pooled sera elicited by the same dose of yeast-derived RBD-dimer potently neutralized live SARS-CoV-2 with NT50 of 16,453 (Fig. 6d) after the third (last) immunization. Thus, we speculate that the immunogenicity of yeast-derived RBD-dimer is very likely comparable to that of CHO-produced RBD-dimer. However, a side-by-side comparison between yeast-derived and CHO-derived RBD vaccines is necessary to draw a solid conclusion.

In this study, the protective efficacy of the vaccines was evaluated in a previously established mouse model of SARS-CoV-2 infection^26,38^. In this model, wild-type (WT) BALB/c mice were inoculated intranasally with adenovirus 5 expressing human ACE2 (Ad5-hACE2) 3 days before live SARS-CoV-2 challenge. Transduction with Ad5-hACE2 could result in high expression of hACE2 receptor in the mouse lung, allowing efficient SARS-CoV-2 replication. Consequently, after live SARS-CoV-2 challenge, very high levels of viral RNA and severe interstitial pneumonia could be detected in Ad5-hACE2-transduced BALB/c mice but not in the WT mice without Ad5-hACE2 transduction^26,38^. In this study, we found that, at 3 days after virus challenge, the viral loads detected in the RBD-monomer- or RBD-dimer-immunized mice were lower than those in the control mice by 40- and 120-folds, respectively (Fig. 6e), demonstrating high protective efficacy of the yeast-produced vaccines. It is noted that no significant weight loss or other clinical symptoms were observed for the vaccinated or control mice in our challenge system. As more rigorous animal models for SARS-CoV-2 infection have been reported, such as K18-transgenic mice or Syrian golden hamsters^39,40^, which upon challenge developed manifestations mimicking clinical symptoms, such as apparent weight loss, respiratory distress, and even death, these animal models can be used to further validate the efficacy of our yeast-produced RBD vaccine candidates in the future.

The emergence of new SARS-CoV-2 variants, such as B.1.1.7 and B.1.351 that carry multiple mutations on the S protein, present a new challenge for SARS-CoV-2 vaccine development. It has been reported that convalescent plasma/sera able to neutralize the original SARS-CoV-2 D614G lineage partially or completely lost neutralization activity toward B.1.351^41^. Recently, Wang et al. also reported that B.1.351 is refractory to neutralization by convalescent plasma and sera from mRNA vaccine recipients with neutralization titers decreasing by ~11–33-fold and ~6.5–8.6-fold, respectively^24^. These data raise a serious concern on the protective efficacy of current vaccines against the predominant circulating SARS-CoV-2 variants. Surprisingly, in the present study, we found that the antisera from mice immunized with yeast-derived RBD-monomer or -dimer could neutralize B.1.1.7 and B.1.351 as efficiently as toward the original vaccine strain in pseudovirus neutralization assays (Fig. 7). Specifically, the geometric mean NT_50_s against the original strain (Wuhan-Hu-1), B.1.1.7, and B.1.351 were 2540, 3592, and 2540, respectively, for the RBD-monomer antisera, and were 6400, 3200, and 2850, respectively, for the RBD-dimer antisera. Our data thus suggest that yeast-derived RBD-based vaccines may provide broad-spectrum protection against infections with SARS-CoV-2 variants.

Collectively, our work shows that *P. pastoris*-produced monomeric and dimeric RBD proteins are able to elicit broadly neutralizing antibodies and long-lasting protective immunity against SARS-CoV-2 infection in mice, demonstrating the proof-of-concept for yeast-derived RBD-based SARS-CoV-2 vaccines. Such a SARS-CoV-2 vaccine technology may be readily transferred to developing countries for local massive production and subsequent deployment, which in turn will help control the SARS-CoV-2 pandemic on a global level.

## Materials and methods

### Cells and viruses

VeroE6 and HEK 293T cells were cultured in DMEM (Gibco, USA) supplemented with 10% fetal bovine serum (FBS; Gibco) at 37 °C. HEK 293F (Thermo Fisher, USA) suspension cells were grown in FreeStyle 293 expression medium (Gibco). HEK 293T cells overexpressing the human ACE2 receptor (293T-hACE2) were generated in a previous study^26^. SARS-CoV-2 clinical strain nCoV-SH01 (GenBank: MT121215.1)^42^ was propagated in VeroE6 cells and virus titers were shown as plaque-forming units (pfu) per mL. All live virus infection experiments were performed in the biosafety level-3 (BSL-3) laboratory of Fudan University.

### Recombinant proteins and antibodies

HEK 293F-expressed RBD with a C-terminal 6× His-tag was generated in a previous study^26^. The extracellular domain (residues Q18 to S740) of human ACE2 fused with human IgG1 Fc (designated hACE2-Fc) was produced in a previous study^26^.

To prepare an antibody for western blotting analysis, anti-RBD polyclonal antibody was generated by immunizing BALB/c mice with recombinant SARS-CoV-2 RBD protein from inclusion bodies of *Escherichia coli* (*E. coli*) in Freund’s adjuvant (Sigma, USA) by using a protocol described in a previous study^43^. In addition, a polyclonal antibody against HEK 293F-expressed RBD was prepared in our previous study^26^. Anti-SARS-CoV-2 MAbs 2H2 and 3C1 were generated in our previous study^26^.

### Construction of yeast expression vectors

To construct recombinant vector for expression of SARS-CoV-2 RBD-monomer in *P. pastoris*, the RBD gene (residues V320 to G550) of SARS-CoV-2 strain Wuhan-Hu-1 (GenBank ID: MN908947.3) was codon-optimized and synthesized. The optimized RBD gene, bearing a 6× His-tag and a stop codon at 3′ end, was inserted into the backbone vector pPink-α-HC (Invitrogen), yielding plasmid pPink-α-HC-RBD. For expression of RBD-dimer in yeast, a series of plasmids were constructed as shown in Fig. 4a. Specially, two RBD gene fragments with variable lengths were fused together with a 5-amino-acid linker (GGGGS) and then cloned into a modified pPink-α-HC vector that contains a 6× His-tag and a stop codon at 3’ end by using the NEBuilder® HiFi DNA Assembly Master Mix (NEB, UK), yielding plasmids pPink-α-HC-RBD-dimer #1 to #4.

### Preparation of recombinant RBD proteins in *P. pastoris*

The plasmids were linearized by *Afl* II digestion and separately transformed into competent PichiaPink™ Strain 1 (Invitrogen) cells by electroporation. After transformation, the yeast cells were plated onto PAD selection plates lacking adenine. To screen high-expression recombinant yeast strains, 10 colonies were randomly picked and subjected to small-scale expression experiment according to the manufacturer’s instructions. The induced culture supernatants were assessed for RBD protein expression by ELISA as described below.

To produce the RBD-monomer and -dimer proteins, the selected yeast clones were separately cultured in 500 mL BMGY medium at 30 °C with shaking until an OD600 of ~10 was reached. The yeast cells were pelleted by centrifugation and then resuspended in 500 mL BMMY medium with 1% methanol. After 48–60 h induction at 30 °C, the culture supernatants were harvested and concentrated to a volume of 50 to 100 mL in binding buffer (0.5 M NaCl, 20 mM Tris, 10 mM imidazole, pH 7.9) using Stirred Cell (Amicon, Millipore, USA) with 5 kDa cut-off membrane. His-tagged RBD proteins were then purified using Ni-nitrilotriacetic acid (NTA) resin (Novagen) according to manufacturer’s instructions. The purified RBD proteins were analyzed by SDS-PAGE (12% acrylamide) and western blotting as described below.

### Screening of high-expression yeast clones by ELISA

The induced culture supernatants from individual yeast clones were analyzed for protein expression by ELISA. Specially, wells of 96-well plates were coated with 25 μL of culture supernatants plus 25 μL of PBS at 37 °C for 2 h. After blocking with 5% milk in PBST, the plates were added with a polyclonal antibody against HEK 293F-expressed RBD (diluted 1:1000 in PBST), followed by incubation at 37 °C for 2 h. After washing, horseradish peroxidase (HRP)-conjugated goat anti-mouse IgG (Sigma, USA) was added and incubated at 37 °C for 1 h. After color development, absorbance at 450 nm was measured.

### Deglycosylation of RBD

Under reducing conditions, induced cell supernatants or purified RBD proteins were denatured at 100 °C for 10 min in glycoprotein denaturing buffer (NEB) and then digested with peptide-N-glycosidase F (PNGase F; NEB) or endo-N-acetylglucosaminidase H (endo H; NEB) at 37 °C for 1 h according to the manufacturer’s instructions. The deglycosylated proteins were analyzed by SDS-PAGE and/or western blotting.

### SDS-PAGE and western blotting analysis

Induced cell supernatants or purified RBD proteins were separated on 12% SDS-PAGE gels and stained with Coomassie blue R-250 or transferred onto polyvinylidene difluoride (PVDF) membranes (Pall). The membranes were probed with a polyclonal antibody against *E. coli*-expressed RBD, followed by HRP-conjugated goat anti-mouse IgG (Sigma).

### ELISA analysis of the binding of RBD proteins to anti-SARS-CoV-2 MAbs

Yeast-derived RBD-monomer, RBD-dimer, and bovine serum albumin (BSA; control) were twofold serially diluted, starting at 8 μg/mL, and added to 96-well ELISA plates, followed by incubation at 4 °C overnight. After blocking, 1 μg/mL of SARS-CoV-2-specific neutralizing MAbs 3C1 or 2H2 was added and incubated at 37 °C for 1 h, followed by wash and incubation with HRP-conjugated goat anti-mouse IgG (Sigma). After color development, absorbance at 450 nm was measured.

### Bio-layer interferometry (BLI) assay

To measure binding affinities of the recombinant RBD proteins to hACE2 receptor, BLI experiments were performed using an Octet Red96 instrument (Pall FortéBio, USA) following manufacturer’s instructions. Briefly, hACE2-Fc protein was diluted to a concentration of 50 μg/mL in kinetics buffer (0.01 M PBS with 0.02% Tween 20 and 0.1% BSA) and was then immobilized to protein A biosensors (Pall FortéBio). The hACE2-Fc-immobilized biosensors were transferred to wells containing RBD- monomer or -dimer protein samples at varying concentrations (2, 4, 8, 16, and 32 nM) for a 500-s association step. Then, the sensors were transferred to kinetics buffer for a 500-s dissociation step. Data analysis was performed using Octet data analysis software version 11.0 (Pall FortéBio).

### SARS-CoV-2 pseudovirus production

Murine leukemia virus (MLV)-based WT or variant SARS-CoV-2 pseudoviruses were generated using a previously reported protocol^26^. Plasmids encoding the full-length S protein of different SARS-CoV-2 strains were generated based on published sequences, including an original (WT) strain Wuhan-Hu-1 (GenBank ID: MN908947.3), the B.1.1.7 variant (GISAID ID: EPI_ISL_601443)^44^, and the B.1.351 variant^45^. Mutations in the B.1.1.7 S protein include ∆H69/∆V70, ∆Y144, N501Y, A570D, D614G, P681H, T716I, S982A, and D1118H^44^. Mutations in the B.1.351 S protein include L18F, D80A, D215G, ∆242 to 244, R246I, K417N, E484K, N501Y, D614G, and A701V^45^.

### Inhibition of pseudovirus entry by RBD proteins

HEK 293T cells stably overexpressing hACE2 (293T-hACE2) were seeded in 96-well white plates at a density of 6000 cells/well and incubated at 37 °C for 20 h. Then, 60 μg/mL of BSA (control), yeast-derived RBD-monomer, or RBD-dimer were added onto the cells and incubated for 1 h. SARS-CoV-2 pseudovirus was then added to the wells and incubated for 12 h. Next, the supernatants were removed, and 200 μL of fresh culture medium was added to each well. After another 48-h culture, luciferase activity was measured using the luciferase assay system (Promega).

### Mouse immunization

All the animal experiments in this study were approved by the Institutional Animal Care and Use Committee at the Institut Pasteur of Shanghai.

In the first experiment, as shown in Fig. 2a, groups of six female BALB/c mice (6–8 weeks old) were immunized intraperitoneally (i.p.) with yeast-derived RBD-monomer protein (50 μg/dose) or PBS formulated with 500 μg of aluminum hydroxide (Alhydrogel, Invivogen, USA) at week 0, and boosted with the same dose at weeks 1 and 3. Blood samples were collected from individual mice at weeks 3, 5, and 9.

In the second experiment, as shown in Fig. 3a, four groups of six female BALB/c mice (6–8 weeks old) were injected i.p. with alum-formulated PBS, 10 μg of RBD formulated with alum, 10 μg of RBD formulated with alum and CpG, or 40 μg of RBD formulated with alum at weeks 0, 2, and 4. Blood samples were taken from individual mice at weeks 3, 6, 10, and 15.

In the third experiment, as shown in Fig. 5a, groups of six adult female BALB/c mice were immunized i.p. with yeast-derived RBD-dimer protein (5 μg/dose) or PBS formulated with alum at weeks 0, 2, and 4. Blood samples were harvested from individual mice at weeks 4, 6, and 21.

In the last experiment, as shown in Fig. 6a, three groups of adult female BALB/c mice (6 mice/group) were immunized i.p. with alum-formulated RBD-monomer, RBD-dimer, or PBS at week 0, 2, and 4. Serum samples were taken at weeks 2, 4, 6, 12, and 22.

### Serum antibody measurement

For titration of RBD-specific antibodies in mouse antisera, 96-well microtiter plates were coated with 25 ng/well of HEK 293F-expressed RBD protein at 4 °C overnight. After blocking, the plates were incubated with 50 μL/well of serially diluted mouse antisera for 2 h at 37 °C, followed by incubation with HRP-conjugated anti-mouse IgG (Sigma). After color development, absorbance at 450 nm was measured. RBD-binding IgG endpoint titer was defined as the highest serum dilution that exhibited >0.1 optical density absorbance units above that of pre-immune serum samples.

For receptor competition assay, microplates were coated at 4 °C overnight with 25 ng/well of HEK 293F-expressed RBD. After blocking, 25 μL/well of serially diluted serum samples were mixed with 25 μL/well (20 ng) of biotinylated ACE2-hFc^26^ and then added to the wells, followed by incubation at 37 °C for 2 h. HRP-conjugated streptavidin (Life Technologies, USA) were added and incubated at 37 °C for 1 h. After washing and color development, absorbance was monitored at 450 nm.

To determine the IgG1/IgG2a ratios, microplates were coated with 25 ng/well of RBD and blocked. Mouse serum samples were diluted 1:10,000, added at 50 μL/well and incubated at 37 °C for 2 h. HRP-conjugated goat anti-mouse IgG1 antibody or HRP-conjugated goat anti-mouse IgG2a antibody (Southern-Biotech, USA) were added and incubated, followed by color development and absorbance measurement. The ratio of IgG1 and IgG2a isotypes was calculated by dividing the OD values of IgG1 by the OD values of IgG2a.

### Pseudovirus neutralization assay

Serum samples were heat-inactivated before neutralization assay. 50 μL/well of twofold serially diluted serum samples and incubated with equal volume of pseudovirus at 37 °C for 1 h. The mixtures were then added to wells in 96-well plates pre-seeded with 293T-hACE2 cells. After incubation at 37 °C for 12 h, the serum/pseudovirus mixtures were removed and the cells were added with fresh culture medium. After another 48-h culture, the cells were lysed and luciferase activity was measured using the luciferase assay system (Promega). The 50% neutralization titer (NT50) was defined as the highest serum dilution at which the relative luminescence units (RLUs) were reduced by 50% compared with virus control wells.

### Live SARS-CoV-2 virus neutralization assay

Authentic SARS-CoV-2 virus neutralization was carried out as previously described^20^. Viral RNA levels in culture supernatants were determined by real-time RT-PCR and viral antigens in cells were detected using immunofluorescence assay as described previously^20^.

### In vivo protection assays

For protection assays, mice were raised in the BSL-3 laboratory of Fudan University and received humane care in compliance with the guidelines of the Animal Research Ethics Board of Fudan University. The mouse model of SARS-CoV-2 infection is based on recombinant adenovirus 5 expressing human ACE2 (Ad5-hACE2)^26^.

To assess the protective efficacy of anti-RBD polyclonal antibody, IgG antibodies (2 mL) were purified from 1.5 mL of anti-PBS or anti-RBD sera using protein G agarose resin 4FF (Yeasen, China) according to manufacturer’s protocol. Groups of WT male BALB/c mice (6–8 weeks old) were inoculated via intranasal route with 5 × 10^10^ virus particles (VP) of Ad5-hACE2 at –3 days post infection (dpi). Next, Ad5-hACE2-transduced mice were injected i.p. with the purified IgG (550 μL/mouse) at –1 dpi and then infected intranasally with 1.15 × 10^5^ PFU of SARS-CoV-2 at day 0.

To assess the protective efficacy of vaccines, all mice immunized with yeast-derived RBD-monomer, RBD-dimer, or PBS were inoculated intranasally with 5 × 10^10^ VP of Ad5-hACE2 at –3 dpi and then infected intranasally with 1.15 × 10^5^ PFU of SARS-CoV-2 at day 0.

For all the challenge assays, the mice were euthanized at 3 dpi and the lungs were collected for determination of viral RNA levels by real-time RT-PCR as described previously^26^.

### Statistics analysis

All statistical analyses were performed using GraphPad Prism software v6.0. Statistical significance was analyzed using Student’s *t*-test.

## Supplementary information

Supplementary Information
