# Supplementary material for: Yeast-produced RBD-based recombinant protein vaccines elicit broadly neutralizing antibodies and durable protective immunity against SARS-CoV-2 infection
Source: Cell Discov. 2021 Aug 18;7:71. doi: 10.1038/s41421-021-00315-9 (PMC8372230; doi:10.1038/s41421-021-00315-9)
Supplement: Supplementary file 1 — Supplementary Information [file 41421_2021_315_MOESM1_ESM.pdf]

Supplementary Information

Yeast-produced RBD-based recombinant protein vaccines elicit broadly neutralizing antibodies and durable protective immunity against SARS-CoV-2 infection

Jinkai Zang[1, #], Yuanfei Zhu[2, #], Yu Zhou[1, #], Chenjian Gu[2, #], Yufang Yi[4, 5, #], Shuxia Wang[1], Shiqi Xu[1], Gaowei Hu[3], Shujuan Du[2], Yannan Yin[1], Yalei Wang[1], Yong Yang[1], Xueyang Zhang[1], Haikun Wang[1], Feifei Yin[4, 5], Chao Zhang[1, *], Qiang Deng[2, *], Youhua Xie[2, 6,*], Zhong Huang[1, *]

[1] CAS Key Laboratory of Molecular Virology & Immunology, Institut Pasteur of Shanghai, Chinese Academy of Sciences, University of Chinese Academy of Sciences, Shanghai, China

[2] Key Laboratory of Medical Molecular Virology (MOE/NHC/CAMS), Department of Medical Microbiology and Parasitology, School of Basic Medical Sciences, Shanghai Institute of Infectious Diseases and Biosecurity, Shanghai Medical College, Fudan University, Shanghai, China

[3] BSL-3 Laboratory of Fudan University, School of Basic Medical Sciences, Shanghai Medical College, Fudan University, Shanghai, China

[4] Key Laboratory of Tropical Translational Medicine of Ministry of Education, Hainan Medical University, Haikou, China

[5] Hainan Medical University-The University of Hong Kong Joint Laboratory of Tropical Infectious Diseases, Hainan Medical University, Haikou, Hainan, China

[6] Children's Hospital, Shanghai Medical College, Fudan University, Shanghai, China

[#] These authors contributed equally: Jinkai Zang[1, #], Yuanfei Zhu[2, #], Yu Zhou[1, #], Chenjian Gu[2, #], Yufang Yi[4, 5, #]

* Corresponding author: Zhong Huang (huangzhong@ips.ac.cn) or Youhua Xie (yhxie@fudan.edu.cn) or Qiang Deng (qdeng@fudan.edu.cn) or Chao Zhang (chaozhang@ips.ac.cn).

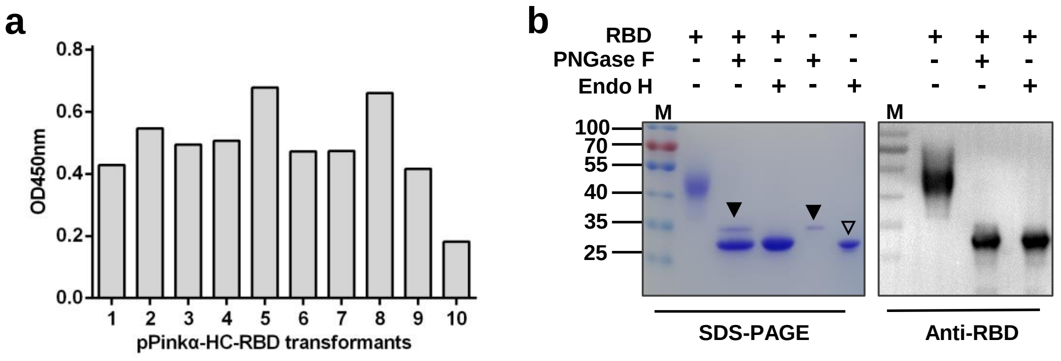

Supplementary Fig. S1 Expression and characterization of SARS-CoV-2 RBD monomer in *Pichia pastoris*. Related to Figure 1. (**a**) ELISA analysis of culture supernatants of pPinkα-HC-RBD vector-transformed yeast clones. A polyclonal antibody against HEK 293F-expressed RBD served as detection antibody. Data are means of two replicate wells. (**b**) Purified monomeric RBD protein was digested with endo H or PNGase F and then subjected to SDS-PAGE (left panel) and western-blotting (right panel) analysis with a polyclonal antibody against *E. coli*-expressed RBD. The PNGase F bands were indicated with filled arrow head. The Endo H bands were indicated with open arrow head. Symbol (+) indicates presence; (-) indicates absence. M, protein marker.

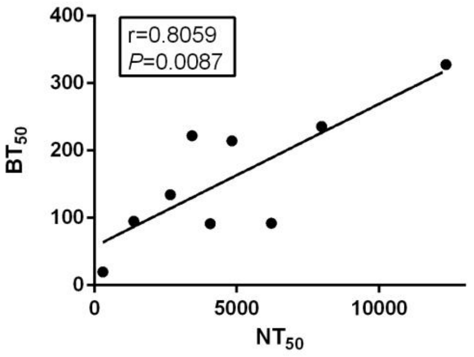

Supplementary Fig. S2 Correlation between the ACE2/RBD binding-inhibition ability and SARS-CoV-2 pseudovirus neutralization potency of anti-RBD sera. Related to Figure 2. BT50 (50% blocking titer) and NT50 values of the individual anti-RBD sera collected at weeks 5 and 9 were used for Pearson correlation coefficient analysis by using GraphPad Prism software.

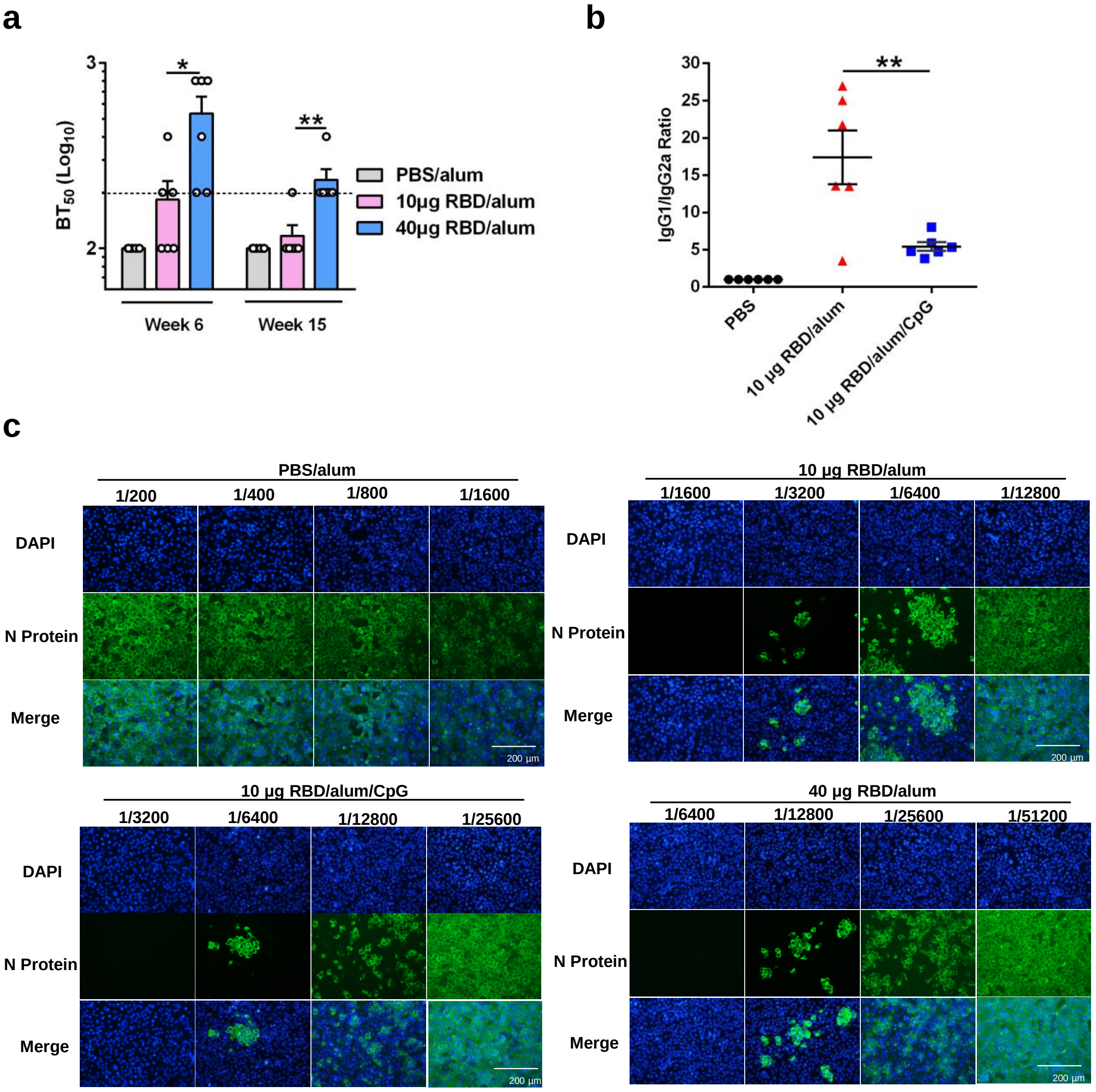

Supplementary Fig. S3 Influence of antigen dose and adjuvant on RBD-induced antibody responses. Related to Figure 3. (**a**) HACE2-binding blockade antibody titers of antisera were measured by ELISA. Blocking titer below 1:200 (the lowest serum dilution; dashed line) was assigned a value of 1:100 for statistical analysis. Each symbol represents one mouse. (**b**) IgG1/IgG2a ratios of anti-RBD antibodies. IgG isotypes of the week-6 serum samples were detected by ELISA. Each symbol represents one mouse. Bars represent the mean ± SEM. Statistical significance was determined by a two-tailed Student‘s t-test and indicated as follows: *, $p < 0.05$, **, $p < 0.01$. (**c**) Neutralizing activity of the pooled week-6 antisera against live SARS-CoV-2 determined by immunofluorescent staining. Bar, 200 μm.

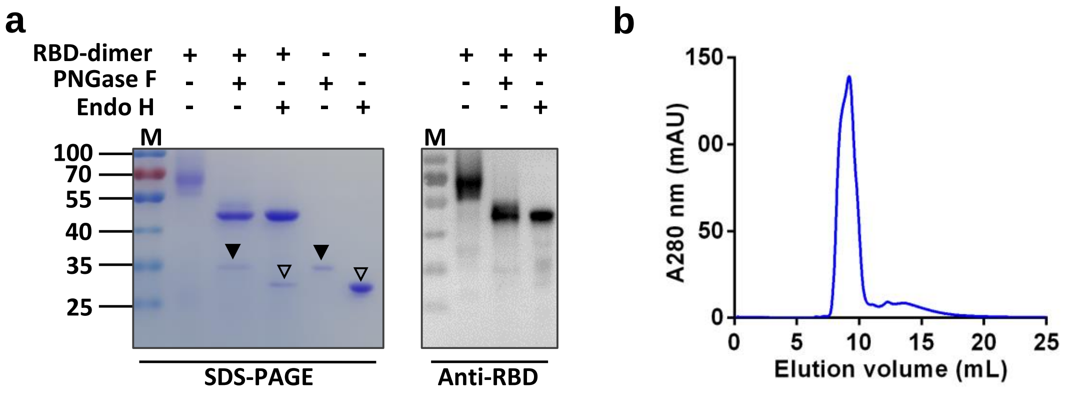

Supplementary Fig. S4 Characterization of purified RBD dimer protein. (**a**) Purified RBD dimer protein was digested with endo H or PNGase F and then subjected to SDS-PAGE (left panel) and western-blotting (right panel) analysis with a RBD-specific polyclonal antibody. Related to Figure 4. The PNGase F bands were indicated with filled arrow head. The Endo H bands were indicated with open arrow head. Symbol (+) indicates presence; (-) indicates absence. M, protein marker. (**b**) Size-exclusion chromatography analysis of the purified SARS-CoV-2 dimer-RBD protein was performed on Superdex 75 increase column.

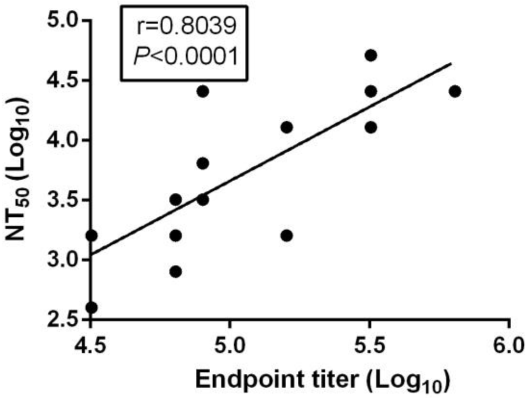

Supplementary Fig. S5 Correlation between RBD binding titer and SARS-CoV-2 pseudovirus neutralization potency of RBD dimer immune sera. Related to Figure 5. NT50 and binding titer values of the individual anti-RBD-dimer sera taken at week 4, 6 and 21 were used for Pearson correlation coefficient analysis using GraphPad Prism software.

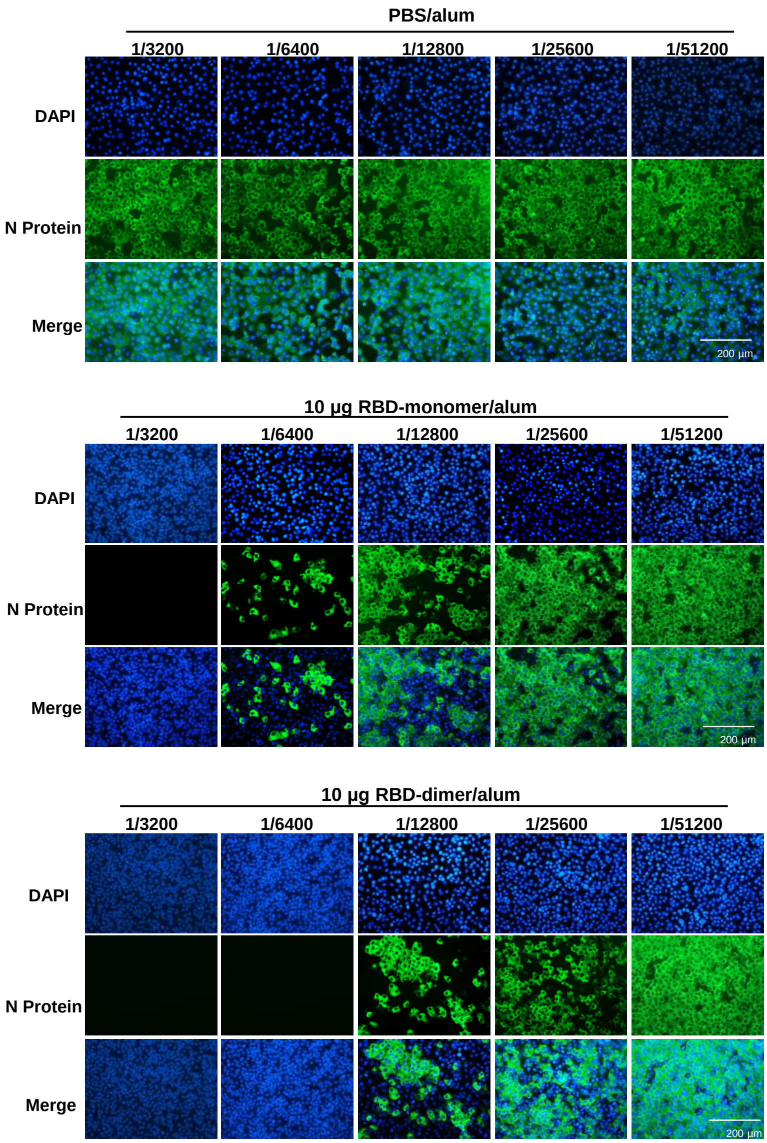

Supplementary Fig. S6 Neutralizing activity of the pooled week-6 antisera against live SARS-CoV-2 measured by immunofluorescence analysis. Related to Figure 6. Bar, 200 µm.
